# Supplementary material for: Subtyping Lung Cancer Using DNA Methylation in Liquid Biopsies
Source: J Clin Med. 2019 Sep 19;8(9):1500. doi: 10.3390/jcm8091500 (PMC6780554; doi:10.3390/jcm8091500)
Supplement: Supplementary file 1 [file jcm-08-01500-s001.pdf]

Supplementary Tables:

Table S1 - Primers used in study group #1 tissue samples and qMSP conditions for each gene.;

Table S2 - Primers and probe sequences used in study group #2 and #3 plasma samples with respective fluorochrome and quencher.;

Table S3 – Associations between clinical stage and *APC*, *HOXA9*, *RARβ2*, and *RASSF1A* methylation levels in study group #1. *P* values obtained by Kruskal–Wallis followed by Mann–Whitney U tests and Bonferroni's correction;

Table S4 – Associations between clinical stage and *APC*, *HOXA9*, *RARβ2*, and *RASSF1A* methylation levels in study group #2. *P* values obtained by Kruskal–Wallis followed by Mann–Whitney U tests and Bonferroni's correction.

Supplementary Figures:

Figure S1. Receiver operating characteristic (ROC) curve of (A) *APC* and (B) *RASSF1A* for LCa detection in plasma samples (study group #2 and study group #3).

Figure S2. Receiver operating characteristic (ROC) curve of (A) *HOXA9* and (B) *RASSF1A* for small-cell lung cancer (SCLC) detection in plasma samples (study group #2).

Figure S3. Receiver operating characteristic (ROC) curve of *HOXA9* for squamous cell carcinoma detection in tissue samples (study group #1).

Supplementary Tables

Table S1 - Primers used in study group #1 tissue samples and qMSP conditions for each gene.

Gene		Sequences	Annealing Temperature	Primer concentration (μM)
β-Actin	Primers	F – 5′ TGG TGA TGG AGG AGG TTT AGT AAG T 3′	60	400
		R – 5′ ACC AAT AAA ACC TAC TCC TCC CTT AA 3′		
APC	Primers	F – 5′ TGT GTT TTA TTG CGG AGT GC 3′	62	300
		R – 5′ CAC ATA TCG ATC ACG TAC GC 3′		
HOXA9	Primers	F – 5′ TAT TTA GTC GGT ATT CGC 3′	60	300
		R – 5′ ACC TCG AAC GCT TCC CAT 3′		
RARβ2	Primers	F – 5′ TCG AGA ACG CGA GCG ATT 3′	60	300
		R – 5′ GAC CAA TCC AAC CGA AAC 3′		
RASSF1A	Primers	F – 5′ AGC GAA GTA CGG GTT TAA TC 3′	60	300
		R – 5′ ACA CGC TCC AAC CGA ATA 3′		

Table S2 - Primers and probe sequences used in study group #2 and #3 plasma samples with respective fluorochrome and quencher.

Gene		Sequences
β-Actin	Primers	F – 5′ TGG TGA TGG AGG AGG TTT AGT AAG T 3′
		R – 5′ ACC AAT AAA ACC TAC TCC TCC CTT AA 3′
	Probe	5′ Cy5 – ACC ACC ACC CAA CAC ACA ATA ACA AAC ACA – QSY 3′
APC	Primers	F – 5′ TGT GTT TTA TTG CGG AGT GC 3′
		R – 5′ CAC ATA TCG ATC ACG TAC GC 3′
	Probe	5′ VIC – CAA TCG ACG AAC TCC CGA C – MGB 3′
HOXA9	Primers	F – 5′ TAT TTA GTC GGT ATT CGC 3′
		R – 5′ ACC TCG AAC GCT TCC CAT 3′
	Probe	5′ FAM –GAA ACT ACC AAA CCG C– MGB 3′
RARβ2	Primers	F – 5′ TCG AGA ACG CGA GCG ATT 3′
		R – 5′ GAC CAA TCC AAC CGA AAC 3′
	Probe	5′ HEX – CTT ACA AAA AAC CTT CCG AAT ACG TTC CGA – Iowa Black RQ-Sp 3′
RASSF1A	Primers	F – 5′ AGC GAA GTA CGG GTT TAA TC 3′
		R – 5′ ACA CGC TCC AACC GA ATA 3′
	Probe	5′ NED – CGG GAG TTG GTA TTC GTT GGG CG – QSY 3′

Table S3 – Associations between clinical stage and *APC*, *HOXA9*, *RARβ2,* and *RASSF1A* methylation levels in study group #1. *P* values obtained by Kruskal–Wallis followed by Mann–Whitney U tests and Bonferroni's correction.

	**Clinical Stage**			
	APC	*HOXA9*	*RARβ2*	*RASSF1A*
I vs II	n.s.	-	n.s.	n.s.
I vs III	n.s.	-	n.s.	n.s.
I vs IV	<0.0001	-	<0.0001	<0.0001
II vs III	n.s.	-	n.s.	n.s.
II vs IV	0.024	-	0.0006	0.018
III vs IV	0.084	-	0.024	0.024
p value Kruskal–Wallis	0.001	n.s.	<0.0001	<0.0001

n.s., not significant

Table S4 – Associations between clinical stage and *APC*, *HOXA9*, *RARβ2,* and *RASSF1A* methylation levels in study group #2. *P* values obtained by Kruskal–Wallis followed by Mann–Whitney U tests and Bonferroni's correction.

	**Clinical Stage**			
	APC	*HOXA9*	*RARβ2*	*RASSF1A*
I vs II	-	n.s.	-	n.s.
I vs III	-	n.s.	-	n.s.
I vs IV	-	n.s.	-	n.s.
II vs III		n.s.		n.s.
II vs IV	-	n.s.	-	n.s.
III vs IV	-	n.s.	-	0.018
p value Kruskal–Wallis	0.073	0.002	n.s.	0.014

n.s., not significant

Supplementary Figures

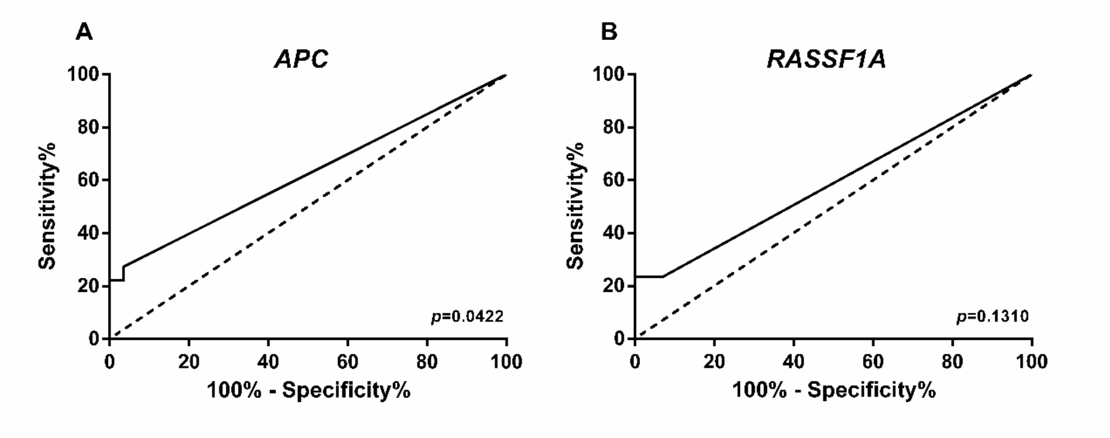

Figure S1. Receiver operating characteristic (ROC) curve of (A) *APC* and (B) *RASSF1A* for LCa detection in plasma samples (study group #2 and study group #3).

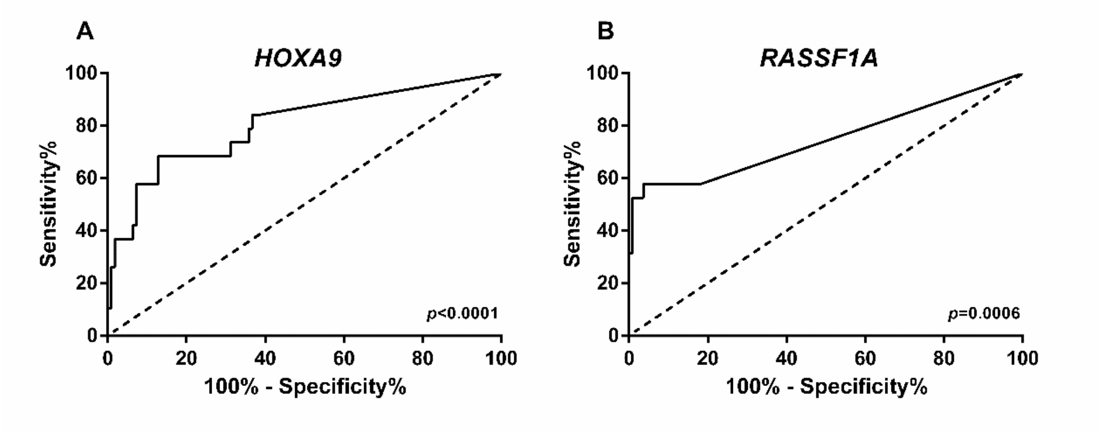

Figure S2. Receiver operating characteristic (ROC) curve of (A) *HOXA9* and (B) *RASSF1A* for small-cell lung cancer (SCLC) detection in plasma samples (study group #2).

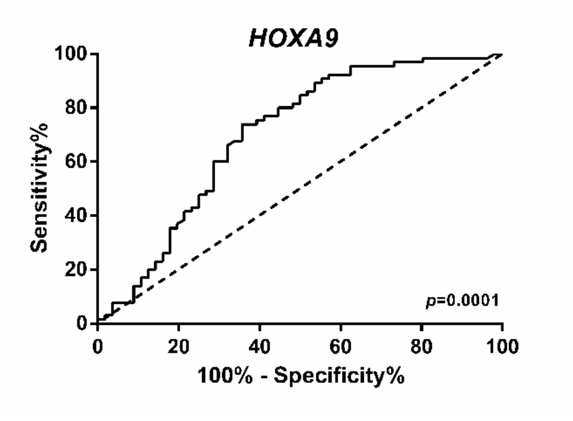

Figure S3. Receiver operating characteristic (ROC) curve of *HOXA9* for squamous cell carcinoma detection in tissue samples (study group #1).
